# Production and Properties of Molybdenum Disulfide/Graphene Oxide Hybrid Nanostructures for Catalytic Applications

**DOI:** 10.3390/nano10091865

**Published:** 2020-09-17

**Authors:** Zuzanna Bojarska, Marta Mazurkiewicz-Pawlicka, Stanisław Gierlotka, Łukasz Makowski

**Affiliations:** 1 Faculty of Chemical and Process Engineering, Warsaw University of Technology, 00-645 Warsaw, Poland; zuzanna.bojarska.dokt@pw.edu.pl (Z.B.); marta.pawlicka@pw.edu.pl (M.M.-P.); 2 Polish Academy of Sciences, Institute of High Pressure Physics UNIPRESS, 01-424 Warsaw, Poland; xray@unipress.waw.pl

**Keywords:** molybdenum disulfide nanoparticles, carbon nanomaterials, impinging jet reactor

## Abstract

Molybdenum disulfide (MoS_2_) can be an excellent candidate for being combined with carbon nanomaterials to obtain new hybrid nanostructures with outstanding properties, including higher catalytic activity. The aim of the conducted research was to develop the novel production method of hybrid nanostructures formed from MoS_2_ and graphene oxide (GO). The nanostructures were synthesized in different weight ratios and in two types of reactors (i.e., impinging jet and semi-batch reactors). Physicochemical analysis of the obtained materials was carried out, using various analytical techniques: particle size distribution (PSD), thermogravimetric analysis (TGA), FT-IR spectroscopy, X-ray diffraction (XRD), and scanning electron microscopy (SEM). Due to the potential application of materials based on MoS_2_ as the catalyst for hydrogen evolution reaction, linear sweep voltammetry (LSV) of the commercial MoS_2_, synthesized MoS_2_ and the obtained hybrid nanostructures was performed using a three-electrode system. The results show that the developed synthesis of hybrid MoS_2_/GO nanostructures in continuous reactors is a novel and facile method for obtaining products with desired properties. The hybrid nanostructures have shown better electrochemical properties and higher onset potentials compared to MoS_2_ nanoparticles. The results indicate that the addition of carbon nanomaterials during the synthesis improves the activity and stability of the MoS_2_ nanoparticles.

## 1. Introduction

The designing and production of new nanostructures with improved properties using modern techniques is a tempting prospect for nanotechnology. Molybdenum disulfide (MoS_2_) is a widely used two dimensional nanomaterial with unique properties [1,2]. MoS_2_ nanoparticles have found various applications such as a dry lubricant, hydrogen evolution reaction (HER) catalyst, hydrodeoxygenation (HDO) catalyst, hydrodesulfurization (HDS) catalyst, in energy storage, and many others [2,3,4,5,6,7,8]. Nanosized MoS_2_ can be an excellent candidate for being combined with carbon nanomaterials (CNMs) to obtain new hybrid nanostructures with outstanding properties, including higher electro- [9,10] and photocatalytic activity [11,12] or improved rheological and tribological properties [13,14].

In recent years, the application of MoS_2_ nanoparticles as the catalyst for hydrogen evolution reactions has generated great interest, due to the search for new renewable energy sources [15,16,17,18]. HER is the cathodic reaction occurring during water splitting (Equations (1) and (2)) [19,20]. Water splitting can lead to a sustainable source of clean hydrogen, which can be stored, used in fuel cells, or in other industrial applications [15,21,22,23]. Catalysts based on noble metals (including platinum) are known as the most effective for HER. Due to their scarcity and high prices, it is necessary to find cheaper alternatives [2,24,25].

(1) $$\text{In}\;\text{alkaline}\;\text{conditions}:\;2H_{2}O+2e^{-}↔2OH^{-}+H_{2}$$

(2) $$\text{In}\;\text{acidic}\;\text{conditions}:\;2H^{+}+2e^{-}\to H_{2}$$

A nontoxic, environmentally friendly semiconductor-nanometric molybdenum disulfide (MoS_2_) seems to be a very promising catalyst for electrocatalytic HER. MoS_2_-based catalysts have a relatively low cost, high chemical stability, and excellent electrocatalytic properties. Therefore, nano-MoS_2_ has attracted enormous research interest as the electrocatalyst for hydrogen evolution reaction [24,26,27,28,29]. Furthermore, nanometric MoS_2_ is the first known two dimensional semiconductor with a bandgap equal to 1.8 eV and an indirect absorbance edge at over 800 nm. Therefore, nanoparticles of molybdenum disulfide may also find an application in photo-electrocatalytic HER [24,30,31,32,33].

Besides, MoS_2_/CNMs hybrid nanomaterials show enhanced catalytic activity from the synergetic effect between carbon nanomaterials and MoS_2_ nanoparticles. This effect has many advantages, including enhancing the number of exposed edges per catalyst volume, increasing active surface area, suppression of charge recombination, improvement of interfacial charge transfer, and increased number of photocatalytic reaction centers [2,12,34,35,36]. Finding a facile and scalable method of production of MoS_2_ nanoparticles deposited on carbon nanomaterials with desired properties (such as nanometric size, narrow particle size distribution, high specific surface area, specific band gap) can be crucial for further development of these materials.

Shi et al. presented a method for synthesizing MoS_2_/graphene hybrid heterostructures with a growth template of graphene-covered copper foil. The chemical vapor deposition (CVD) of MoS_2_ on the graphene surface ensures the formation of monocrystalline hexagonal flakes with a typical lateral size from several hundred nanometers to several micrometers [37]. Li et al. synthesized MoS_2_ on reduced graphene oxide (rGO) structures via a microwave-assisted reduction in graphite oxide in an aqueous solution of a MoS_2_ precursor. The obtained materials showed improved photocatalytic performance, due to the lower recombination of electron-hole pair and enhanced light absorption [38]. Song et al. presented a synthesis method of MoS_2_ nanosheets on rGO surface by a one-step hydrothermal growth technique. MoS_2_/rGO materials showed excellent electrochemical performance for Na-ion batteries [39]. Similarly, Murugan et al. deposited MoS_2_ nanosheets on rGO by an in situ hydrothermal method, resulting in MoS_2_/rGO materials consisting of a few-layered MoS_2_ structures with abundantly exposed edges stacked onto rGO [40]. Whereas Kumar et al. have synthesized MoS_2_ nanoparticles deposited on graphene oxide (GO) and rGO, obtaining materials with promising electrocatalytic properties for HER [41]. Therefore, direct formation of MoS_2_ on graphene surface exhibits great potential towards HER catalysis and other advanced electrochemical applications. However, the above-mentioned methods have many drawbacks that prevent the commercialization of these materials. CVD and microwave-assisted methods are very expensive and not scalable, in turn, hydrothermal methods are characterized by a lack of process control, quality and repeatability of the manufactured materials. Thus, a novel method of producing MoS_2_/CNMs hybrid nanostructures with the use of continuous flow reactors by a wet chemical synthesis is proposed in this paper [42,43,44]. The synthesis of hybrid nanostructures carried out in continuous flow reactors (such as impinging jet reactors) allows an almost instantaneous mixing of reagent streams at the molecular level, which has an important role in obtaining products of high quality and repeatability. Due to the good mixing conditions, materials obtained in the impinging jet reactor might show better dispersion of MoS_2_ particles on the carbon surface, smaller particle size, and a lower tendency to agglomerate, which has a significant impact in advanced applications, such as catalysis. Moreover, this technique allows for the continuous production of materials with the desired properties [42,45,46,47,48,49]. The aim of the conducted research was to examine if impinging jet reactors can be used for preparation of MoS_2_/GO hybrid structures with enhanced properties compared to the more commonly used semi-batch reactor. Due to better mixing conditions, better scalability, and better process control, this method should result in high-quality MoS_2_/GO nanostructures with desired properties. In this paper the materials were synthesized in different ratios of MoS_2_ and GO and in different types of reactors (i.e., impinging jet and semi-batch reactors). The properties of the obtained materials were characterized using different analytical techniques, such as particle size distribution (PSD), thermogravimetric analysis (TGA), Fourier-transform infrared (FT-IR) spectroscopy, X-ray diffraction (XRD) and scanning electron microscopy (SEM). Furthermore, the electrochemical properties towards a hydrogen evolution reaction were tested. The results have shown that the use of continuous flow reactors allows to obtain MoS_2_/GO hybrid structures with desirable properties (such as nanometric size, narrow particle size distribution, enhanced catalytic activity and stability) and better electrochemical activity compared to MoS_2_ nanoparticles.

## 2. Materials and Methods

### 2.1. Graphene Oxide

Graphene oxide was synthesized by a modified Hummers’ method as described in [50,51]. GO was obtained through the oxidation of graphite powder using 98% sulfuric acid H_2_SO_4_ (Chempur, Piekary Śląskie, Poland) and potassium permanganate KMnO_4_ (Chempur, Piekary Śląskie, Poland) in the presence of sodium nitrate NaNO_3_ (Sigma-Aldrich, Saint Louis, MI, USA). Generally, for 10 g of graphite, 230 mL of H_2_SO_4_, 30 g of KMnO_4_ and 4.7 g of NaNO_3_ were used. The chemicals were added in an ice bath, where the temperature was kept below 10 °C. Next, the mixture was stirred for 2 h with the control of the temperature not exceeding 30 °C. After the process, 100 mL of water was added and the temperature increased up to 100 °C. Further, the mixture was treated with 10 mL of 30% hydrogen peroxide H_2_O_2_ (Sigma-Aldrich, Saint Louis, MI, USA), and at the end of the process filtrated in a special filtration system with ceramic membranes and exfoliated by ultrasonic treatment. The final product was in the form of an 0.34 wt% aqueous suspension of graphene oxide.

### 2.2. Molybdenum Disulfide

Molybdenum disulfide was produced through the wet chemical synthesis in the impinging jet reactor with tangential (V-type) geometry as described in [42,43,44]. The synthesis was carried out using ammonium heptamolybdate tetrahydrate (NH_4_)_6_Mo_7_O_24_∙4H_2_O (HMA) (Chempur, Piekary Śląskie, Poland) and ammonium sulfide (NH_4_)_2_S (AS) (Sigma-Aldrich, Saint Louis, MI, USA). Citric acid C_6_H_8_O_7_ (CA) (Stanlab, Lublin, Poland) was used as a reducing agent in this reaction. HMA and CA with Mo:CA molar ratio of 1:2 were dissolved in water at 90 °C and mixed for at least 30 min. 20 wt.% solution of AS was diluted, maintaining the molar ratio of Mo:AS of 1:2. Both prepared solutions were filtrated using a set with three filtration steps (1 µm; 0.45 µm; 0.22 µm). The reaction was carried out at 25 °C. The obtained suspension was purified through three times centrifugation (RCF 21390) for 1 h in Sigma 3–18 K Centrifuge (Osterode am Harz, Germany). After each centrifugation the supernatant was removed from the precipitate, and the sample was re-dispersed in deionized (DI) water for 15 min in an ultrasonic bath. The purified precipitate was dried at 50 °C until the dark-brown powder was received. Further purification and crystallization were conducted through annealing at 850 °C for 1 h in argon flow in the vacuum retort furnace.

### 2.3. MoS_2_/GO Hybrid Nanostructures

MoS_2_/GO hybrid nanostructures were obtained by a wet chemical synthesis with the same reagents as in the case of MoS_2_ synthesis, i.e., HMA, CA, AS. The reaction was carried out in two types of reactors: a semi-batch reactor and an impinging jet reactor. The structures were synthesized in different, assumed weight ratios of MoS_2_:GO, such as 10:1; 30:1; 50:1. The corresponding GO masses were calculated based on the amount of MoS_2_ produced during the synthesis reaction (Equations (3) and (4)) assuming 100% efficiency [42].

(3) $${\left(NH_{4}\right)}_{6}Mo_{7}O_{24}\;+21{\left(NH_{4}\right)}_{2}S\;\overset{H_{3}O^{+}}{\to }\;7MoS_{2}↓+7S↓+24H_{2}O+48NH_{3}↑$$

(4) $${\left(NH_{4}\right)}_{2}S+2C_{6}H_{8}O_{7}\to H_{2}S↑+2C_{6}H_{7}O_{7}NH_{4}$$

#### 2.3.1. Synthesis in the Semi-Batch Reactor

**Figure 1:**
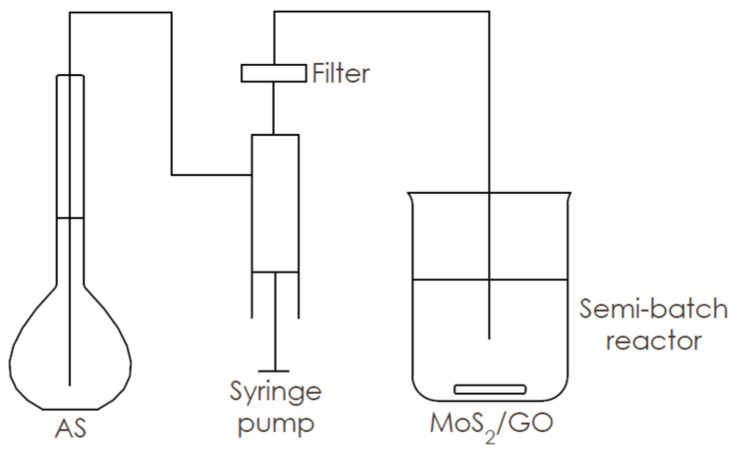
Synthesis of Molybdenum disulfide (MoS_2_)/ graphene oxide (GO) in the semi-batch reactor.

0.706 g of HMA and 1.537 g CA were dissolved in ~10 mL of DI water at 90 °C and mixed for at least 30 min with Mo:CA molar ratio of 1:2. After cooling to room temperature, an appropriate amount (Table 1) of 0.34 wt.% aqueous suspension of GO was added to the mixture, filled up to 20 mL with DI water and dispersed in an ultrasonic bath for 30 min. A total of 2.734 mL of 20 wt.% AS solution was diluted in 20 mL of DI water. Subsequently, the reagents were thermostated at 25 °C. AS solution was supplied to the reactor with a flow rate of 20 mL/min with GO + HMA + CA suspension, previously passed through a filter set with three filtration steps (1 µm; 0.45 µm; 0.22 µm). The product was a dark-brown suspension of MoS_2_/GO. A scheme of the used system with the semi-batch reactor is presented in Figure 1.

#### 2.3.2. Synthesis in the Impinging Jet Reactor

**Figure 2:**
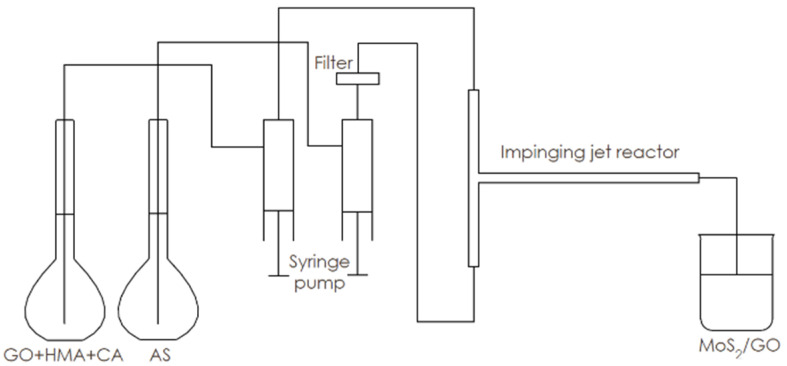
Synthesis of MoS_2_/GO in the impinging jet reactor.

The substrates were prepared by the same procedure as in the 2.3.1 stage. One of the inlets of the reactor was supplied with AS solution and another with GO + HMA + CA suspension. The reactor works in a continuous flow rate of 20 mL/min, using a syringe pump. The reaction was carried out at 25 °C. The product, as in the semi-batch procedure, was a dark-brown suspension of MoS_2_/GO. A scheme of the used system with the impinging jet reactor is presented in Figure 2.

Table 1 contains the amounts of reagents used during the synthesis, both in the semi-batch reactor and the impinging jet reactor.

### 2.4. Purification

To remove impurities, the obtained suspensions were centrifuged (RCF 21390) for 1 h in Sigma 3–18 K Centrifuge (Osterode am Harz, Germany). The supernatant containing impurities from the samples was removed, and the precipitates were re-dispersed in DI water using the ultrasonic bath for the next 15 min. This procedure was repeated three times. The precipitates were dried at 50 °C for 24 h until the dark powders were received. Recent studies report that amorphous molybdenum disulfide may have better catalytic properties than its crystalline form [52,53]. The aim of the conducted annealing is to remove sulfur from the obtained hybrid nanostructures. The boiling point of sulfur is around 445 °C [54], and to ensure the appropriate temperature, the annealing was carried out at a temperature higher by 100 °C, due to the fact that at higher temperatures, the carbon material may be decomposed. The powders were placed in the vacuum retort furnace and annealed at 550 °C for 1 h in an argon flow. The final products were in the form of powders with graphite color.

### 2.5. Structural Characterization

A thorough physicochemical analysis of the synthesized materials was carried out, using various analytical techniques. The direct product after the reaction was characterized by LS 13 320 Laser Diffraction Particle Size Analyzer from Beckman Coulter Life Sciences (Brea, CA, USA). Characteristic particle size L_10_ of the synthesized products was calculated using moments of the PSD (Equation (5)).

(5) $$m_{k}=\overset{\infty }{\underset{0}{\int }}L^{k}n\left(L\right)dL\;\;\;\;\;\;\;\text{for}\;k=1,\;2,\ldots$$

For the final products thermogravimetric analysis, FT-IR spectroscopy, X-ray powder diffraction, and scanning electron microscopy performed. TGA of the hybrid nanostructures and MoS_2_ was conducted from 30 °C to 1600 °C with 20 °C/min heating rate in 30 mL/min argon flow using TGA/DSC (Thermal Gravimetric Analysis/Differential Scanning Calorimetry) 3 + analyzer from Mettler Toledo (Columbus, OH, USA). FT-IR was carried out using Nicolet iS10 spectrometer from Thermo Scientific (Waltham, MA, USA) in the transmittance mode. For this measurement, a small amount of each hybrid material and GO was mixed with KBr powder (0.5 wt.%) and pressed into pellets. The spectrum was collected in the range 500–4000 cm^−1^ with a 4 cm^−1^ resolution. The XRD analysis was carried out using Panalytical X’Pert Pro (Malvern, United Kingdom) diffractometer with a copper lamp CuKα1. The average size of the crystallites was calculated using Scherrer equation. The SEM images were taken using field emission scanning electron microscope (FE-SEM) Hitachi S5500 (Chiyoda, Tokyo, Japan) with a resolution of 0.4 nm. Two modes: the secondary electrons (SE) mode and the bright-field scanning transmission electron microscopy (BF-STEM) were used. The samples were prepared by applying a few drops of the hybrid nanostructures’ suspension in acetone on the TEM copper grid with carbon film.

### 2.6. Electrochemical Measurements

To check the potential application of the synthesized MoS_2_ and hybrid nanostructures as catalysts for HER, electrochemical measurements were performed and compared with reference MoS_2_ from Sigma-Aldrich (Saint Louis, Missouri, United States) (90 nm, 99%). To prepare the catalyst ink, 6 mg of the material was added to 0.5 mL of terpineol (≥96%, Sigma-Aldrich (Saint Louis, MO, USA)) and dispersed using ultrasounds for 60 min. The suspension was spin-coated (500 rpm for 30 s) onto the fluorine-doped tin oxide (FTO) glass substrate. The prepared electrodes were dried at a hot plate at 80 °C. Subsequently, electrodes were placed in the vacuum retort furnace FCF 2R Czylok (Jastrzębie-Zdrój, Poland) and annealed at 550 °C for 2 h in an argon atmosphere to increase the ductility of the material [34]. Linear sweep voltammetry (LSV) was performed using a three-electrode system on Keithley 2450 Source Meter/Unit (Cleveland, OH, USA). The reference electrode was Ag/AgCl and platinum wire was used as the counter electrode. The aqueous electrolyte was 0.5 M H_2_SO_4_. Measurements were taken for each sample 5 times.

## 3. Results and Discussion

**Figure 3:**
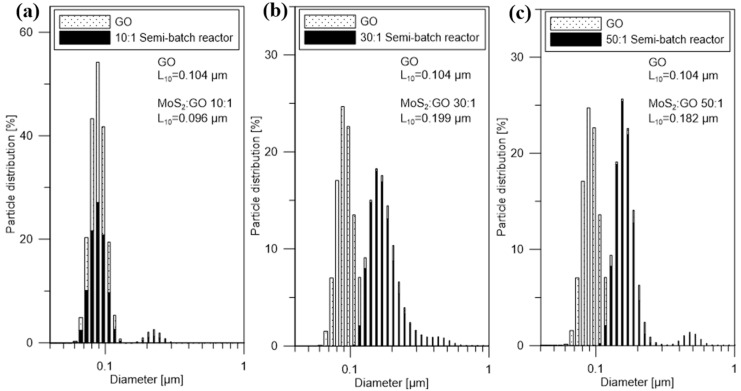
Particle size distribution profiles by number of particles of direct products of the reaction in the semi-batch system for MoS_2_/GO ratio (**a**) 10:1 (**b**) 30:1 (**c**) 50:1, and GO just before the reaction.

Figure 3 shows the particle size distributions of GO (just before the reaction) and MoS_2_/GO (direct products of the reaction in a semi-batch reactor) with different weight ratios. The characteristic particle sizes L_10_ of GO and MoS_2_/GO (10:1; 30:1; 50:1) were 0.104, 0.096, 0.199, 0.182 µm, respectively. The addition of carbon nanomaterials to the reaction environment allows primary heterogeneous nucleation to occur. MoS_2_ particles precipitated and continued to grow on graphene flakes, obtaining hybrid nanostructures. In samples 30:1 and 50:1 PSDs show that the characteristic particle size increased due to the coverage of GO flake by the synthesized MoS_2_ particles. In the case of the 10:1 sample, there was practically no change in the characteristic size. For this sample, the largest amount of GO was used, meaning it had the biggest amount of nucleation centers. The lack of change in this sample may indicate that the synthesized MoS_2_ particles were so small and evenly distributed on the GO surface that their formation did not affect the characteristic particle size. Compared to the precipitation of the pure MoS_2_ in the semi-batch reactor (as described in [42]) with the same process parameters (25 °C, initial Mo concentration of 0.2 mol/dm^3^), obtained hybrid nanostructures are more stable and have a lower tendency to agglomerate. PSDs of the hybrid nanostructures do not show the presence of agglomerates, and in the case of pure MoS_2_ PSD shows a distinct peak of agglomerates with the characteristic particle size equal to 1.4 µm [42].

**Figure 4:**
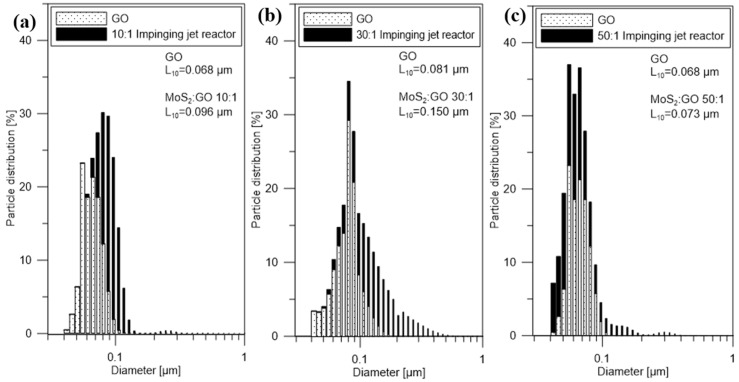
Particle size distribution profiles by number of particles of direct products of the reaction in the impinging jet reactor for MoS_2_/GO ratio (**a**) 10:1 (**b**) 30:1 (**c**) 50:1 and GO just before the reaction.

Figure 4 shows the particle size distributions of GO (just before the reaction) and MoS_2_/GO (direct products of the reaction in the impinging jet reactor) with different weight ratios. The same as in the case of a semi-batch reactor, PSDs show that the characteristic particle size L_10_ increased due to the covering of GO flakes by MoS_2_ particles. The characteristic particle sizes L_10_ of MoS_2_/GO (10:1; 30:1; 50:1) were 0.096, 0.150, 0.073 µm, respectively. The use of the impinging jet reactor in the MoS_2_/GO synthesis allowed to obtain smaller particle sizes compared to the materials from the semi-batch reactor, due to the better mixing conditions. Similarly, as in the semi-batch procedure, obtained hybrid nanostructures are more stable and have a lower tendency to agglomerate.

**Figure 5:**
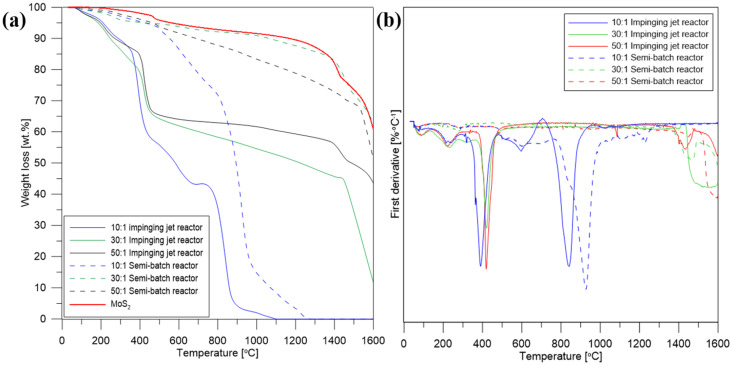
(**a**) Thermogravimetric analysis and (**b**) derivative thermogravimetric of the hybrid nanostructures and MoS_2._

Thermogravimetric analysis of the purified and annealed materials is shown in Figure 5a and the derivative thermogravimetric analysis (DTG) in Figure 5b. A small initial weight loss at 100 °C for all samples corresponds to the evaporation of adsorbed water. Further weight loss around 200 °C for hybrid structures obtained in the impinging jet reactor is attributed to the removal of oxygen-containing functional groups from graphene oxide. For these samples another distinct weight decrease is visible around 400 °C, due to the oxidation of MoS_2_ to MoO_3_, resulting probably from the oxygen released during the degradation of GO. However, the materials synthesized in the semi-batch reactor do not show weight loss at this temperature range, hence the amount of oxygen from the GO structure wasn’t sufficient for the oxidation of MoS_2_ to MoO_3_ [55,56]. Further gradual weight loss of the rest of the samples corresponds to the removal of stable oxygen functional groups. Due to worse mixing conditions in the semi-batch reactor, it is likely that MoS_2_ particles were not only deposited on the carbon surface, but some were formed as a result of homogeneous nucleation. Therefore, some of the carbon material was not completely covered with MoS_2_ and due to its hydrophilic properties, some of GO was removed during purification, hence its lower content in the samples compared to the materials from the impinging jet reactor. TG analysis of the synthesized samples confirmed that the synthesis carried out in the impinging jet reactor allows to obtain stable hybrid nanostructures based on MoS_2_ and carbon nanomaterials.

**Figure 6:**
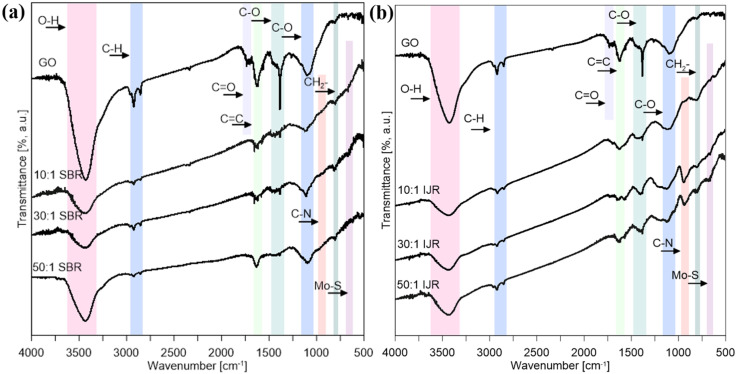
Fourier-transform infrared (FT-IR) spectra of GO and MoS_2_/GO synthesized in (**a**) the semi-batch reactor (**b**) the impinging jet reactor.

FT-IR spectra of GO, the structures synthesized in the semi-batch reactor and in the impinging jet reactor are shown in Figure 6. GO has shown characteristic peaks associated with O–H stretching at 3200–3600 cm^−1^, C–H stretching at 2850–2960 cm^−1^, carbonyl C=O stretching at 1710–1740 cm^−1^, C=C stretching about 1625 cm^−1^, C–O stretching vibration of alkoxy group at 1300–1420 cm^−1^, C–O stretching vibration of carboxyl groups at 1050–1150 cm^−1^, and CH_2_− at 760 cm^−1^ [57]. The decrease in the peak intensity of the oxygen-rich functional groups present in the synthesized nanostructures suggests a reduction in GO. In the case of the hybrid nanostructures, a peak associated with C–N stretching at 900–1000 cm^−1^ can be observed, suggesting that GO was additionally functionalized by HMA during the preparation of GO + HMA + CA suspension in the ultrasonic bath. For the samples obtained in the impinging jet reactor the signal of C–N stretching is stronger (Figure 6b). It might suggest that due to the high intensity of mixing in the area of collision of streams an additional functionalization is also taking place in the reactor. This functionalization can positively affect the electrochemical and photocatalytic properties of the obtained materials, due to the possibility of creating a p-n junction between the N–GO and MoS_2_ (which is a p-type semiconductor). Furthermore, a weak peak at 600 cm^−1^ is assigned to the Mo–S stretching vibration presented in MoS_2_-based materials [58].

**Figure 7:**
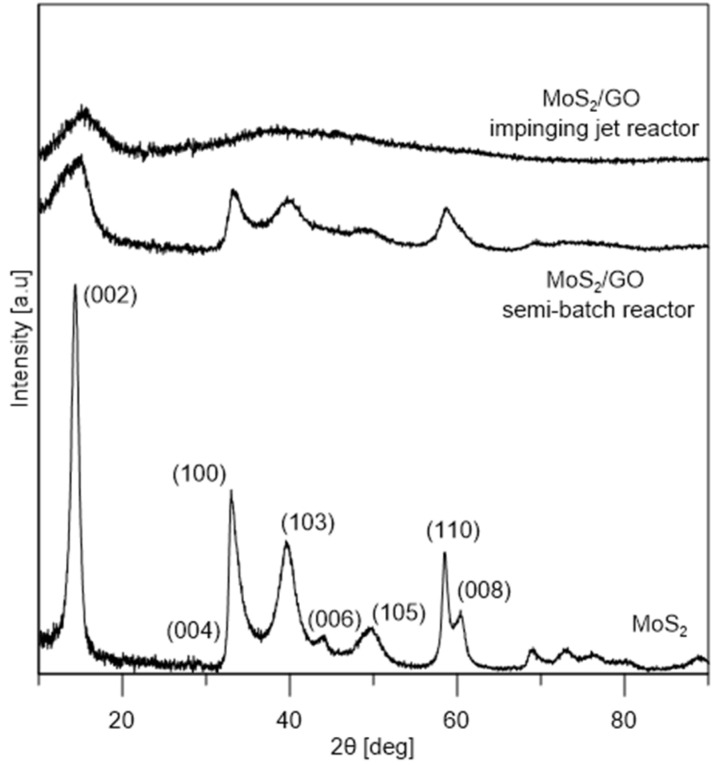
X-ray diffraction (XRD) patterns of MoS_2_, MoS_2_/GO synthesized in the semi-batch reactor and in the impinging jet reactor.

XRD analysis (Figure 7) confirmed that synthesized MoS_2_ is crystalline with a hexagonal crystal structure 2H–MoS_2_. The average crystallite size is 9 nm in c-direction and 12 nm in a-direction. In the case of MoS_2_/GO from the semi-batch reactor the average size of MoS_2_ crystallites is 3.1 nm in c-direction and 7.4 nm in a-direction, and from impinging jet reactor it is ~1.6 nm in c-direction. The addition of GO to the synthesis of MoS_2_ allowed to obtain smaller crystallites. This is due to the fact that graphene oxide has many functional groups that can serve as nucleation sites for MoS_2_ particles. In the impinging jet reactor the obtained particles are smaller, which can be attributed to the better mixing conditions. Due to the lower annealing temperature of MoS_2_/GO, the hybrid nanostructures show lower crystallinity.

**Figure 8:**
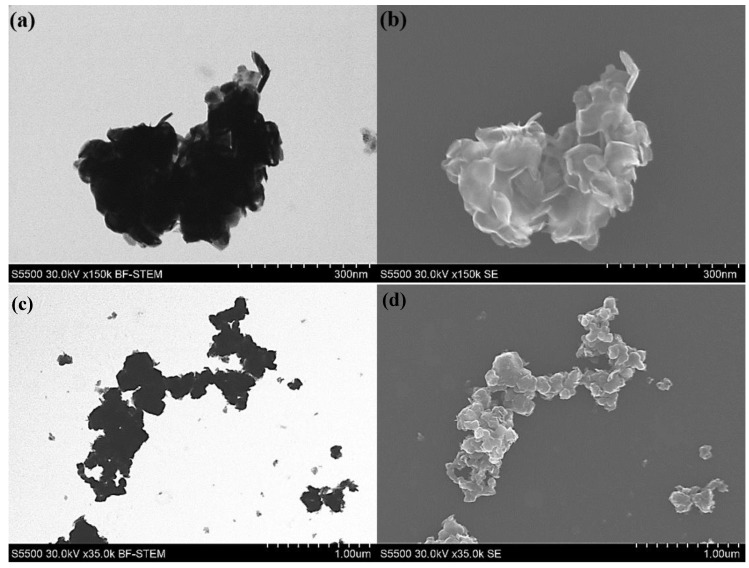
Images of synthesized MoS_2_ particles in (**a**,**c**) the bright-field scanning transmission electron microscopy (BF-STEM) and (**b**,**d**) in secondary electrons (SE) modes.

SEM images of the synthesized MoS_2_ are shown in Figure 8. As can be seen, synthesized molybdenum disulfide has a crystal structure with a layered surface and in the form of flakes. SEM images showed that MoS_2_ particles tend to agglomerate obtaining micrometer size.

**Figure 9:**
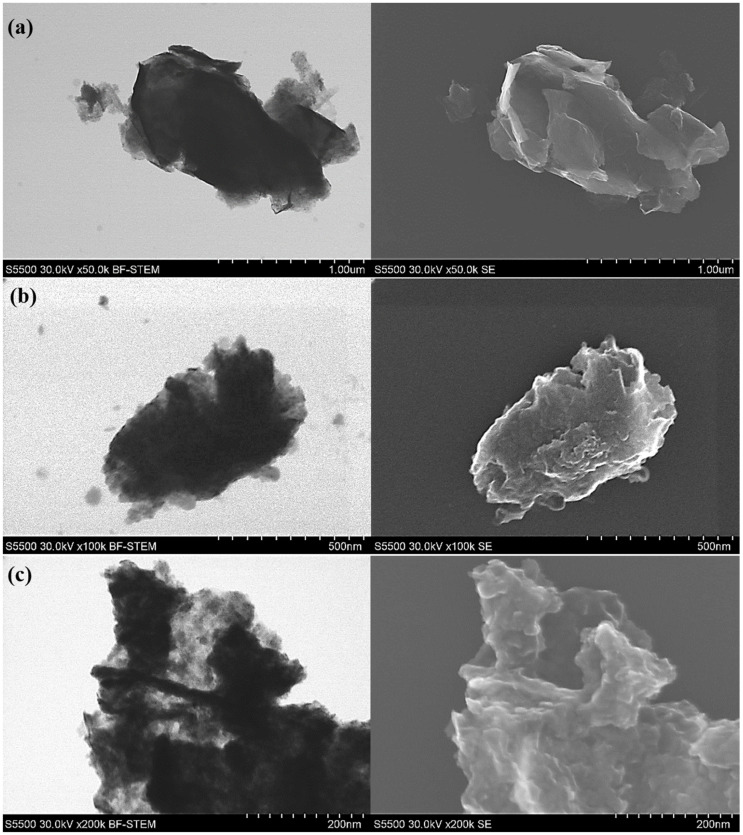
Images of MoS_2_/GO form in the semi-batch reactor in weight ratios of (**a**) 10:1, (**b**) 30:1, (**c**) 50:1 in SE and BF-STEM modes.

Figure 9 shows SEM images of the obtained hybrid nanostructures in the semi-batch reactor. SEM images have confirmed the XRD results that MoS_2_ nanoparticles on GO are not fully crystalline. Nanoparticles of MoS_2_ are attached to the carbon surfaces, forming shapes close to GO flakes. With increasing MoS_2_ content, the hybrid structures resemble pure molybdenum disulfide. Furthermore, the hybrid nanostructures exhibit a lower tendency to agglomerate, comparing to pure MoS_2_ particles.

**Figure 10:**
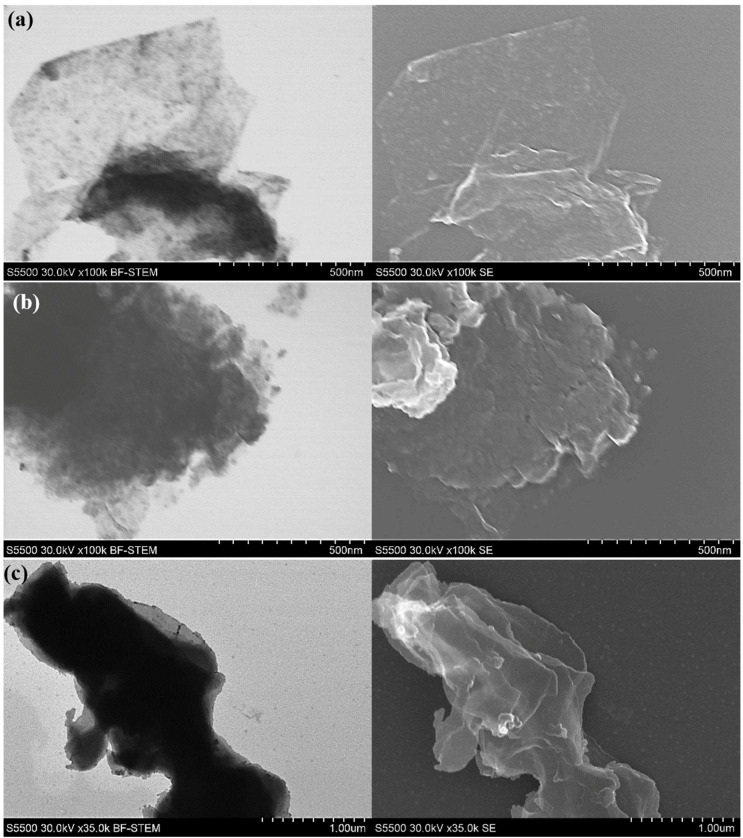
Images of MoS_2_/GO form in the impinging jet reactor in weight ratios of (**a**) 10:1, (**b**) 30:1, (**c**) 50:1 in SE and BF-STEM modes.

Figure 10 shows SEM images of the obtained hybrid nanostructures in the impinging jet reactor. As with the particles from the semi-batch reactor, the MoS_2_ nanoparticles are not fully crystalline. MoS_2_ nanoparticles can be observed on the GO flakes’ surface (Figure 10a). This structure is different than for the samples obtained in the semi-batch reactor, confirming the XRD results that the particles are smaller. In the pictures it can be seen that MoS_2_ particles attach to GO surface, changing their morphology. In both cases (semi-batch and impinging jet reactor) MoS_2_ seems to be agglomerated (except in Figure 10a, where small MoS_2_ nanoparticles are evenly distributed on the GO surface) and no big differences between the two synthesis methods can be observed. During the synthesis of MoS_2_/GO hybrid structures two processes can occur: heteronucleation, where MoS_2_ nanoparticles will form on GO surface and homonucleation, where MoS_2_ will form in the solution. SEM and PSD results suggest that most of the MoS_2_ particles tend to form on GO flakes, meaning that the formation of MoS_2_ nanoparticles is mainly through heteronucleation.

**Figure 11:**
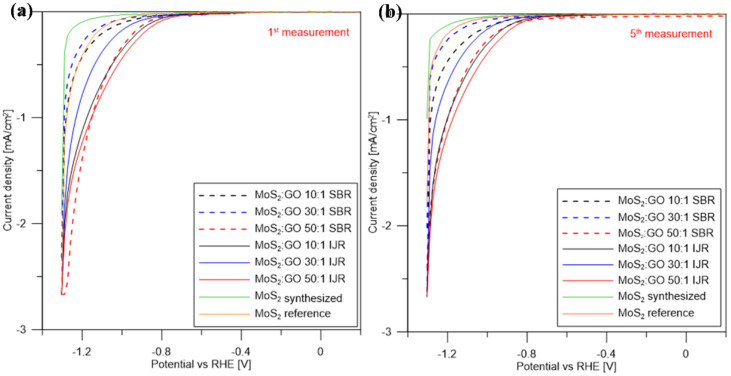
Linear sweep voltammograms of MoS_2_ and MoS_2_/GO performed using three electrodes system (Platinum wire as counter electrode and Ag/AgCl as reference electrode) (**a**) the first measurement, (**b**) the fifth measurement.

Figure 11a shows the linear sweep voltammetry of the obtained samples. The polarization curves of synthesized and commercial MoS_2_ have an onset potential (potential with current density ~0 mA/cm^2^) of −0.63 V, −0.54 V, respectively, and show a sharp decrease in the current density as the potential decreases. In the case of the hybrid nanostructures obtained in the semi-batch reactor (10:1; 30:1; 50:1), the onset potential was −0.40 V, −0.48 V, −0.43 V, respectively, and for the samples from the impinging jet reactor (10:1; 30:1; 50:1) it was −0.39 V, −0.39 V, −0.36 V, respectively. The addition of GO increases the onset potential, possibly due to the formation of smaller MoS_2_ nanoparticles exhibiting more active sites than pure MoS_2_. Another reason for this effect is because carbon flakes serve as conducting support, causing enhanced electrical conductivity and improving the interfacial charge transfer as well as suppression of charge recombination. The 50:1 ratio (both from the impinging jet reactor and semi-batch reactor) exhibits the best catalytic performance. Probably, this ratio allows for uniform precipitation of MoS_2_ on the carbonaceous surface and obtaining nano-sized particles (PSD results). The hybrid nanostructures synthesized in the impinging jet reactor have shown better electrochemical properties, due to the better mixing conditions, providing better dispersion of MoS_2_ particles on the GO surface. Another explanation for this result might be connected to additional functionalization of GO with nitrogen groups during the synthesis of the hybrid structures, which can possibly create a p-n junction between the N-GO and MoS_2_, enhancing the catalytic properties. The LSV curves and onset potential are similar, indicating that the use of impinging jet reactor allows to obtain products with repeatable properties. Figure 11b shows the fifth measurement of LSV. As can be seen, obtained polarization curves for MoS_2_ particles change during the measurements, while for the hybrid nanostructures they are repeatable, hence the addition of GO improves electrochemical stability of MoS_2_ particles.

## 4. Conclusions

The hybrid nanostructures based on molybdenum disulfide and graphene oxide can be successfully obtained in the semi-batch reactor and the impinging jet reactor. Due to the better mixing conditions, materials obtained in the impinging jet reactor have shown better dispersion of MoS_2_ particles on the carbon surface, smaller particle size, less tendency to agglomerate, and higher carbon material contents. During preparation, GO was additionally functionalized by HMA, confirmed by FT-IR measurements, which can be observed especially in the samples from the impinging jet reactor, probably due to the high intensity of mixing in the area of collision of streams. This functionalization can positively affect the electrochemical and photocatalytic properties of the materials obtained, due to the possibility of creating a p-n junction between carbon and MoS_2_ particles. The hybrid nanostructures obtained in the impinging jet reactor have shown better electrochemical properties and higher onset potentials. The results indicated that addition of carbon nanomaterials during the synthesis improves the activity and stability of the MoS_2_.

## Figures and Tables

**Table 1:** The weight of reagents used. HMA: ammonium heptamolybdate tetrahydrate; AS: ammonium sulfide; CA: Citric acid.

MoS_2_/GO Ratio	10:1	30:1	50:1
Volume of reagents’ solution [mL]	20		
20 wt.% AS mass [mL]	2.734		
HMA mass [g]	0.706		
CA mass [g]	1.537		
0.34 wt.% GO mass [g]	12.556	4.186	2.512
